# Bis(μ_2_-2-phen­oxy­propionato-κ^2^ *O*:*O*′)bis­[(1,10-phenanthroline-κ^2^ *N*,*N*′)bis­(2-phen­oxy­propionato-κ^2^ *O*,*O*′)ytterbium(III)]

**DOI:** 10.1107/S1600536811036105

**Published:** 2011-09-14

**Authors:** Jin-Bei Shen, Jia-Lu Liu, Guo-Liang Zhao

**Affiliations:** a College of Chemistry and Life Sciences, Zhejiang Normal University, Jinhua 321004, Zhejiang, People’s Republic of China; b Zhejiang Normal University Xingzhi College, Jinhua, Zhejiang 321004, People’s Republic of China

## Abstract

In the centrosymmetric binuclear title complex, [Yb_2_(C_9_H_9_O_3_)_6_(C_12_H_8_N_2_)_2_], the two Yb(III) ions are linked by two 2-phen­oxy­propionate (*L*) groups in a bidentate bridging mode. Each Yb^III^ ion is eight-coordinated by two O atoms from two bridging *L* ligands, four O atoms from two chelating *L* groups and two N atoms from one chelating phen mol­ecule in a distorted YbN_2_O_6_ dodeca­hedral geometry.

## Related literature

For background to phen­oxy­alkanoic acids, see: Markus & Buser (1997 ▶). For a related Yb complex, see: Lu *et al.* (1999 ▶). For compounds with the same formula type but monoclinic symmetry, see: Shen *et al.* (2011*a* ▶) for Tb; Shen *et al.* (2011*b* ▶) for Pr; Shen *et al.* (2011*c* ▶) for Dy; Shen *et al.* (2011*d* ▶) for La; Shen *et al.* (2011*e* ▶) for Ho; Shen *et al.* (2011*f* ▶) for Gd; Shen *et al.* (2011*g* ▶) for Ce.
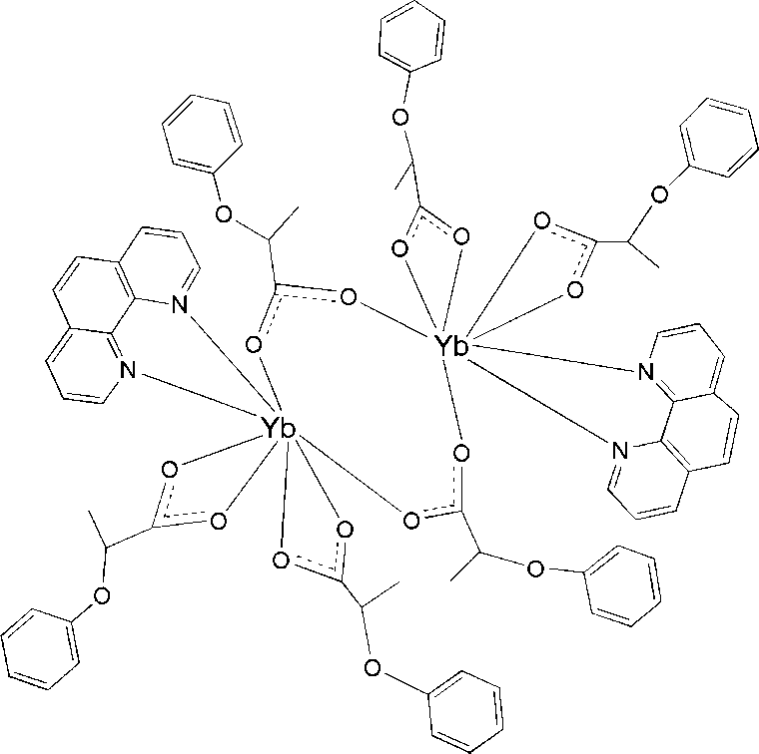


## Experimental

### 

#### Crystal data

[Yb_2_(C_9_H_9_O_3_)_6_(C_12_H_8_N_2_)_2_]
*M* *_r_* = 1697.46
Triclinic, 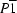

*a* = 11.3577 (4) Å
*b* = 12.2091 (5) Å
*c* = 14.1438 (6) Å
α = 99.111 (2)°
β = 91.089 (2)°
γ = 114.320 (2)°
*V* = 1756.94 (12) Å^3^
*Z* = 1
Mo *K*α radiation
μ = 2.72 mm^−1^
*T* = 296 K
0.32 × 0.20 × 0.06 mm

#### Data collection

Bruker APEXII CCD diffractometer
Absorption correction: multi-scan (*SADABS*; Sheldrick, 1996 ▶) *T*_min_ = 0.524, *T*_max_ = 0.849
22731 measured reflections
6187 independent reflections
5246 reflections with *I* > 2σ(*I*)
*R*_int_ = 0.039

#### Refinement

*R*[*F* ^2^ > 2σ(*F* ^2^)] = 0.030
*wR*(*F* ^2^) = 0.066
*S* = 1.02
6187 reflections
460 parameters
H-atom parameters constrained
Δρ_max_ = 1.00 e Å^−3^
Δρ_min_ = −1.03 e Å^−3^

### 

Data collection: *APEX2* (Bruker, 2006 ▶); cell refinement: *SAINT* (Bruker, 2006 ▶); data reduction: *SAINT*; program(s) used to solve structure: *SHELXS97* (Sheldrick, 2008 ▶); program(s) used to refine structure: *SHELXL97* (Sheldrick, 2008 ▶); molecular graphics: *XP* in *SHELXTL* (Sheldrick, 2008 ▶) and *DIAMOND* (Brandenburg, 2006 ▶); software used to prepare material for publication: *SHELXL97*.

## Supplementary Material

Crystal structure: contains datablock(s) I. DOI: 10.1107/S1600536811036105/wm2528sup1.cif

Structure factors: contains datablock(s) I. DOI: 10.1107/S1600536811036105/wm2528Isup2.hkl

Additional supplementary materials:  crystallographic information; 3D view; checkCIF report

## Figures and Tables

**Table 1:** Selected bond lengths (Å)

Yb1—O7	2.209 (2)
Yb1—O8^i^	2.266 (3)
Yb1—O1	2.340 (3)
Yb1—O4	2.360 (3)
Yb1—O5	2.369 (2)
Yb1—O2	2.403 (3)
Yb1—N2	2.457 (3)
Yb1—N1	2.482 (3)

Symmetry code: (i) 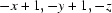.

**Fig. 1.**
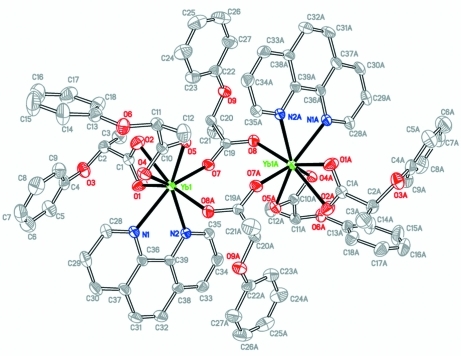
The molecular structure of the title complex, showing the atom-labelling scheme. Displacement ellipsoids are drawn at the 30% probability level.

**Fig. 2.**
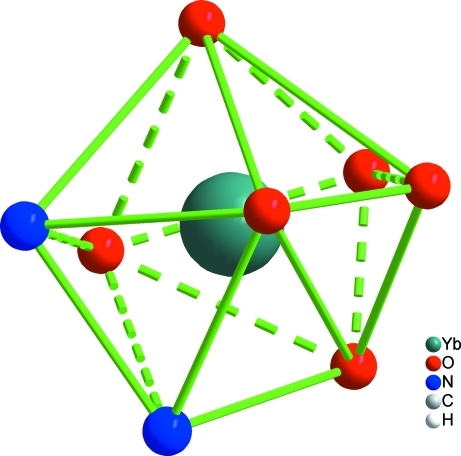
The coordination environment of the Yb(III).

## supplementary crystallographic information

### Comment

The group of phenoxyalkanoic acids includes a considerable number of important herbicides. The desired biological activity is largely dependent on the length of the carbon chain of the alkanoic acid, the nature of the phenoxy group, and the position of its attachment to the carbon chain (Markus & Buser, 1997). The structures of 2-phenoxypropionic acid (H*L*) complexes coupled with their special functionality catched our interest. Here, we describe the Yb^III^ title complex, (I).

The structure of complex (I) is shown in Fig. 1 and the coordination environment of Yb(III) is shown in Fig. 2. The dimeric title compound (I) is centrosymmetric and is comprised of six *L* anions and two phenanthroline ligands. The *L* ligands are coordinated to the Yb^III^ ions in two different modes: chelating and bridging with a Yb—Yb separation of 5.1470 (3) Å. The two Yb(III) ions are linked by two *L* groups through their bidentate bridging modes. Each Yb(III) ion is coordinated to eight atoms, two of which are oxygen atoms from the bridging carboxylates, four oxygen atoms from the bidentate chelating carboxylate groups, and by two nitrogen atoms from a 1,10-phenanthroline molecule. The analysis of structural features indicates that the Yb(III) ion adopts a distorted dodecahedral geometry (Fig. 2), a coordination geometry that is relatively seldom reported for lanthanide carboxylate complexes (Lu *et al.*, 1999). The Yb—O distances are all within the range 2.209 (2)–2.403 (3) Å, and the Yb—N distances rang from 2.457 (3)–2.482 (3) Å, all of which are within the range of those of other eight-coordinated Yb^III^ complexes with carboxylic donor ligands and 1,10-phenanthroline (Lu *et al.*, 1999).

In contrast to the lighter congeners, the Yb(III) complex adopts triclinic symmetry and the metal atom shows coordination number of eight instead of nine. For isoformular compounds with monoclinic symmetry, see: For Tb (Shen *et al.*, 2011*a*), for Pr (Shen *et al.*, 2011*b*), for Dy (Shen *et al.*, 2011*c*), for La (Shen *et al.*, 2011*d*), for Ho (Shen *et al.*, 2011*e*), for Gd (Shen *et al.*, 2011*f*), for Ce (Shen *et al.*, 2011*g*).

### Experimental

Reagents and solvents used were of commercially available quality and without purified before use. 2-Phenoxypropionic acid (1.5 mmol), Yb(NO_3_)_3_^.^6H_2_O (0.5 mmol) and 1,10-phenanthroline (0.5 mmol) were dissolved in 20 ml enthanol, then 10 ml water were added to the above solution. The mixed solution was stirred for 12 h at room temperature. Finally, the deposit was filtered off and the colourless solution was kept in the open air. Colourless crystals were obtained after several days.

### Refinement

The structure was solved by direct methods and successive Fourier difference synthesis. The H atoms bonded to C and N atoms were positioned geometrically and refined using a riding model [aliphatic C—H =0.96 Å (*U*_iso_(H) = 1.5*U*_eq_(C)), aromatic C—H = 0.93 Å (*U*_iso_(H) = 1.2*U*_eq_(C))].

### Crystal data

**Table:**

[Yb_2_(C_9_H_9_O_3_)_6_(C_12_H_8_N_2_)_2_]	*Z* = 1
*M**_r_* = 1697.46	*F*(000) = 850
Triclinic, *P*1	*D*_x_ = 1.604 Mg m^−^^3^
Hall symbol: -P 1	Mo *K*α radiation, λ = 0.71073 Å
*a* = 11.3577 (4) Å	Cell parameters from 8204 reflections
*b* = 12.2091 (5) Å	θ = 1.5–25.0°
*c* = 14.1438 (6) Å	µ = 2.72 mm^−^^1^
α = 99.111 (2)°	*T* = 296 K
β = 91.089 (2)°	Block, colourless
γ = 114.320 (2)°	0.32 × 0.20 × 0.06 mm
*V* = 1756.94 (12) Å^3^	

### Data collection

**Table:**

Bruker APEXII CCD diffractometer	6187 independent reflections
Radiation source: fine-focus sealed tube	5246 reflections with *I* > 2σ(*I*)
graphite	*R*_int_ = 0.039
phi and ω scans	θ_max_ = 25.0°, θ_min_ = 1.5°
Absorption correction: multi-scan (*SADABS*; Sheldrick, 1996)	*h* = −13→13
*T*_min_ = 0.524, *T*_max_ = 0.849	*k* = −14→14
22731 measured reflections	*l* = −15→16

### Refinement

**Table:**

Refinement on *F*^2^	Primary atom site location: structure-invariant direct methods
Least-squares matrix: full	Secondary atom site location: difference Fourier map
*R*[*F*^2^ > 2σ(*F*^2^)] = 0.030	Hydrogen site location: inferred from neighbouring sites
*wR*(*F*^2^) = 0.066	H-atom parameters constrained
*S* = 1.02	*w* = 1/[σ^2^(*F*_o_^2^) + (0.0359*P*)^2^] where *P* = (*F*_o_^2^ + 2*F*_c_^2^)/3
6187 reflections	(Δ/σ)_max_ = 0.001
460 parameters	Δρ_max_ = 1.00 e Å^−^^3^
0 restraints	Δρ_min_ = −1.03 e Å^−^^3^

### Special details

**Table:**

Geometry. All e.s.d.'s (except the e.s.d. in the dihedral angle between two l.s. planes) are estimated using the full covariance matrix. The cell e.s.d.'s are taken into account individually in the estimation of e.s.d.'s in distances, angles and torsion angles; correlations between e.s.d.'s in cell parameters are only used when they are defined by crystal symmetry. An approximate (isotropic) treatment of cell e.s.d.'s is used for estimating e.s.d.'s involving l.s. planes.
Refinement. Refinement of *F*^2^ against ALL reflections. The weighted *R*-factor *wR* and goodness of fit *S* are based on *F*^2^, conventional *R*-factors *R* are based on *F*, with *F* set to zero for negative *F*^2^. The threshold expression of *F*^2^ > σ(*F*^2^) is used only for calculating *R*-factors(gt) *etc*. and is not relevant to the choice of reflections for refinement. *R*-factors based on *F*^2^ are statistically about twice as large as those based on *F*, and *R*- factors based on ALL data will be even larger.

### Fractional atomic coordinates and isotropic or equivalent isotropic displacement parameters (Å^2^)

**Table:**

	*x*	*y*	*z*	*U*_iso_*/*U*_eq_	
Yb1	0.321843 (14)	0.387600 (14)	0.104334 (12)	0.03526 (7)	
O1	0.2116 (3)	0.4766 (3)	0.2049 (2)	0.0605 (9)	
O2	0.3679 (3)	0.4528 (3)	0.2756 (2)	0.0598 (8)	
O3	0.1030 (3)	0.4796 (3)	0.3790 (2)	0.0604 (8)	
O4	0.3356 (2)	0.2126 (2)	0.1446 (2)	0.0497 (7)	
O5	0.5181 (2)	0.3719 (2)	0.14106 (19)	0.0427 (6)	
O7	0.4588 (2)	0.5738 (2)	0.08986 (19)	0.0445 (7)	
O9	0.7307 (3)	0.8066 (2)	0.2317 (2)	0.0530 (7)	
O6	0.4646 (3)	0.1021 (2)	0.2351 (2)	0.0520 (7)	
N1	0.0969 (3)	0.2250 (3)	0.0782 (2)	0.0397 (8)	
N2	0.1801 (3)	0.4252 (3)	−0.0073 (2)	0.0385 (8)	
C1	0.2746 (4)	0.4822 (3)	0.2793 (3)	0.0424 (10)	
C17	0.3960 (6)	0.2501 (6)	0.4604 (4)	0.0785 (16)	
H17A	0.4034	0.3258	0.4920	0.094*	
C28	0.0536 (4)	0.1309 (4)	0.1234 (3)	0.0484 (10)	
H28A	0.1087	0.1272	0.1710	0.058*	
C8	0.0119 (6)	0.1528 (5)	0.3729 (4)	0.0816 (16)	
H8A	0.0491	0.0990	0.3795	0.098*	
C7	−0.1206 (6)	0.1093 (5)	0.3570 (4)	0.0780 (16)	
H7A	−0.1729	0.0263	0.3521	0.094*	
C19	0.5677 (3)	0.6629 (3)	0.1040 (3)	0.0334 (8)	
C16	0.3422 (6)	0.1508 (7)	0.5030 (4)	0.102 (2)	
H16A	0.3139	0.1592	0.5639	0.123*	
C4	0.0358 (4)	0.3546 (4)	0.3711 (3)	0.0541 (11)	
C20	0.5966 (4)	0.7492 (3)	0.2009 (3)	0.0434 (10)	
H20A	0.5496	0.7027	0.2492	0.052*	
C6	−0.1743 (6)	0.1883 (6)	0.3485 (4)	0.0882 (17)	
H6A	−0.2640	0.1595	0.3382	0.106*	
C9	0.0911 (5)	0.2769 (5)	0.3792 (4)	0.0661 (13)	
H9A	0.1808	0.3059	0.3888	0.079*	
C12	0.5823 (5)	0.1475 (4)	0.1017 (3)	0.0613 (12)	
H12A	0.6322	0.1066	0.1216	0.092*	
H12B	0.6354	0.2117	0.0694	0.092*	
H12C	0.5089	0.0900	0.0586	0.092*	
C13	0.4271 (4)	0.1270 (4)	0.3251 (3)	0.0519 (11)	
C18	0.4401 (5)	0.2394 (5)	0.3698 (3)	0.0639 (13)	
H18A	0.4774	0.3072	0.3403	0.077*	
C5	−0.0967 (5)	0.3113 (5)	0.3551 (4)	0.0743 (15)	
H5A	−0.1341	0.3650	0.3488	0.089*	
C10	0.4573 (4)	0.2661 (3)	0.1569 (3)	0.0394 (9)	
C32	−0.1525 (4)	0.2498 (5)	−0.1287 (4)	0.0656 (14)	
H32A	−0.2082	0.2559	−0.1746	0.079*	
C14	0.3728 (6)	0.0264 (5)	0.3684 (4)	0.0841 (17)	
H14A	0.3656	−0.0495	0.3375	0.101*	
C25	0.9109 (6)	0.6397 (6)	0.3733 (4)	0.0799 (17)	
H25A	0.9531	0.6052	0.4077	0.096*	
C24	0.7826 (6)	0.5756 (5)	0.3430 (4)	0.0777 (16)	
H24A	0.7376	0.4977	0.3567	0.093*	
C26	0.9779 (5)	0.7534 (6)	0.3538 (4)	0.0844 (18)	
H26A	1.0659	0.7957	0.3740	0.101*	
C2	0.2398 (4)	0.5281 (4)	0.3769 (3)	0.0493 (11)	
H2A	0.2781	0.5040	0.4280	0.059*	
C15	0.3293 (7)	0.0391 (7)	0.4574 (5)	0.117 (3)	
H15A	0.2910	−0.0287	0.4867	0.141*	
C39	0.0575 (3)	0.3367 (3)	−0.0342 (3)	0.0394 (9)	
C38	−0.0265 (4)	0.3479 (4)	−0.1024 (3)	0.0485 (11)	
C3	0.2877 (5)	0.6651 (4)	0.3944 (4)	0.0704 (14)	
H3A	0.2655	0.6923	0.4561	0.106*	
H3B	0.2479	0.6881	0.3452	0.106*	
H3C	0.3803	0.7022	0.3931	0.106*	
C34	0.1389 (4)	0.5425 (4)	−0.1136 (3)	0.0550 (12)	
H34A	0.1693	0.6142	−0.1386	0.066*	
C11	0.5356 (4)	0.2010 (3)	0.1886 (3)	0.0446 (10)	
H11A	0.6109	0.2602	0.2320	0.054*	
C36	0.0142 (3)	0.2313 (3)	0.0110 (3)	0.0394 (9)	
C30	−0.1523 (4)	0.0386 (4)	0.0319 (3)	0.0557 (12)	
H30A	−0.2338	−0.0258	0.0151	0.067*	
C35	0.2173 (4)	0.5238 (4)	−0.0471 (3)	0.0469 (10)	
H35A	0.3009	0.5844	−0.0295	0.056*	
C22	0.7855 (4)	0.7426 (4)	0.2753 (3)	0.0496 (11)	
C31	−0.1925 (4)	0.1492 (5)	−0.0890 (3)	0.0639 (14)	
H31A	−0.2737	0.0859	−0.1095	0.077*	
C37	−0.1128 (4)	0.1377 (4)	−0.0160 (3)	0.0516 (11)	
C33	0.0169 (4)	0.4542 (4)	−0.1415 (3)	0.0542 (12)	
H33A	−0.0370	0.4647	−0.1861	0.065*	
C29	−0.0720 (4)	0.0362 (4)	0.1028 (3)	0.0551 (12)	
H29A	−0.0996	−0.0274	0.1374	0.066*	
C23	0.7186 (5)	0.6259 (4)	0.2919 (3)	0.0609 (13)	
H23A	0.6316	0.5814	0.2692	0.073*	
C27	0.9167 (4)	0.8060 (5)	0.3046 (4)	0.0684 (14)	
H27A	0.9628	0.8838	0.2910	0.082*	
C21	0.5524 (5)	0.8474 (4)	0.1927 (4)	0.0710 (15)	
H21A	0.5703	0.9013	0.2537	0.106*	
H21B	0.4608	0.8110	0.1739	0.106*	
H21C	0.5978	0.8930	0.1452	0.106*	
O8	0.6489 (3)	0.6912 (2)	0.04486 (19)	0.0453 (7)	

### Atomic displacement parameters (Å^2^)

**Table:**

	*U*^11^	*U*^22^	*U*^33^	*U*^12^	*U*^13^	*U*^23^
Yb1	0.02867 (9)	0.03739 (10)	0.03569 (11)	0.00996 (7)	0.00405 (7)	0.00639 (7)
O1	0.0591 (19)	0.097 (2)	0.0397 (18)	0.0519 (19)	−0.0014 (15)	−0.0004 (16)
O2	0.0611 (19)	0.089 (2)	0.0456 (19)	0.0481 (18)	0.0087 (15)	0.0106 (16)
O3	0.0534 (18)	0.0599 (19)	0.071 (2)	0.0267 (16)	0.0227 (16)	0.0109 (16)
O4	0.0355 (15)	0.0439 (15)	0.066 (2)	0.0119 (13)	−0.0028 (13)	0.0142 (14)
O5	0.0335 (13)	0.0398 (15)	0.0534 (18)	0.0121 (12)	0.0092 (12)	0.0127 (13)
O7	0.0409 (15)	0.0359 (14)	0.0478 (17)	0.0080 (13)	−0.0015 (12)	0.0066 (12)
O9	0.0450 (16)	0.0472 (16)	0.0534 (19)	0.0086 (14)	−0.0024 (14)	0.0034 (14)
O6	0.0615 (18)	0.0459 (16)	0.055 (2)	0.0254 (15)	0.0140 (15)	0.0166 (14)
N1	0.0318 (16)	0.0412 (18)	0.042 (2)	0.0104 (15)	0.0073 (14)	0.0094 (16)
N2	0.0314 (16)	0.0454 (19)	0.0349 (19)	0.0125 (15)	0.0051 (14)	0.0068 (15)
C1	0.038 (2)	0.040 (2)	0.048 (3)	0.0152 (19)	0.0079 (19)	0.0087 (19)
C17	0.090 (4)	0.099 (4)	0.057 (4)	0.054 (4)	0.005 (3)	0.003 (3)
C28	0.042 (2)	0.047 (2)	0.055 (3)	0.015 (2)	0.014 (2)	0.015 (2)
C8	0.103 (5)	0.073 (4)	0.074 (4)	0.043 (4)	0.016 (3)	0.011 (3)
C7	0.083 (4)	0.064 (3)	0.074 (4)	0.017 (3)	0.029 (3)	0.015 (3)
C19	0.034 (2)	0.0298 (19)	0.036 (2)	0.0125 (17)	−0.0005 (18)	0.0078 (17)
C16	0.106 (5)	0.128 (6)	0.058 (4)	0.035 (5)	0.033 (3)	0.012 (4)
C4	0.060 (3)	0.054 (3)	0.045 (3)	0.020 (2)	0.021 (2)	0.010 (2)
C20	0.035 (2)	0.043 (2)	0.045 (3)	0.0106 (18)	0.0050 (18)	0.0042 (19)
C6	0.071 (4)	0.087 (4)	0.093 (5)	0.021 (3)	0.012 (3)	0.012 (4)
C9	0.067 (3)	0.068 (3)	0.069 (3)	0.031 (3)	0.016 (3)	0.017 (3)
C12	0.067 (3)	0.058 (3)	0.070 (3)	0.034 (3)	0.025 (3)	0.016 (2)
C13	0.051 (3)	0.061 (3)	0.048 (3)	0.027 (2)	0.008 (2)	0.011 (2)
C18	0.078 (3)	0.071 (3)	0.055 (3)	0.041 (3)	0.009 (3)	0.017 (3)
C5	0.063 (3)	0.069 (3)	0.092 (4)	0.026 (3)	0.018 (3)	0.019 (3)
C10	0.037 (2)	0.038 (2)	0.041 (2)	0.0151 (19)	0.0068 (17)	0.0024 (18)
C32	0.037 (2)	0.090 (4)	0.062 (3)	0.021 (3)	−0.004 (2)	0.010 (3)
C14	0.103 (4)	0.069 (4)	0.074 (4)	0.025 (3)	0.037 (3)	0.025 (3)
C25	0.102 (5)	0.093 (4)	0.059 (4)	0.062 (4)	−0.013 (3)	−0.004 (3)
C24	0.093 (4)	0.069 (3)	0.073 (4)	0.039 (3)	−0.009 (3)	0.006 (3)
C26	0.060 (3)	0.104 (5)	0.085 (4)	0.043 (3)	−0.019 (3)	−0.015 (4)
C2	0.048 (2)	0.061 (3)	0.043 (3)	0.028 (2)	0.009 (2)	0.005 (2)
C15	0.138 (6)	0.105 (6)	0.081 (5)	0.017 (5)	0.048 (4)	0.029 (4)
C39	0.0292 (19)	0.050 (2)	0.037 (2)	0.0172 (18)	0.0051 (17)	−0.0026 (19)
C38	0.031 (2)	0.071 (3)	0.042 (3)	0.022 (2)	0.0021 (18)	0.005 (2)
C3	0.068 (3)	0.066 (3)	0.063 (3)	0.022 (3)	0.007 (3)	−0.010 (3)
C34	0.055 (3)	0.068 (3)	0.053 (3)	0.031 (2)	0.010 (2)	0.025 (2)
C11	0.041 (2)	0.040 (2)	0.052 (3)	0.0152 (19)	0.0049 (19)	0.011 (2)
C36	0.0314 (19)	0.043 (2)	0.039 (2)	0.0126 (17)	0.0082 (17)	0.0019 (18)
C30	0.033 (2)	0.056 (3)	0.063 (3)	0.006 (2)	0.013 (2)	0.002 (2)
C35	0.037 (2)	0.054 (3)	0.048 (3)	0.014 (2)	0.0050 (19)	0.016 (2)
C22	0.054 (3)	0.052 (3)	0.037 (2)	0.023 (2)	−0.003 (2)	−0.007 (2)
C31	0.031 (2)	0.078 (3)	0.061 (3)	0.006 (2)	−0.006 (2)	−0.001 (3)
C37	0.032 (2)	0.057 (3)	0.053 (3)	0.010 (2)	0.008 (2)	0.000 (2)
C33	0.046 (2)	0.080 (3)	0.046 (3)	0.036 (2)	0.002 (2)	0.011 (2)
C29	0.046 (2)	0.047 (2)	0.068 (3)	0.014 (2)	0.024 (2)	0.014 (2)
C23	0.062 (3)	0.056 (3)	0.060 (3)	0.023 (2)	−0.005 (2)	0.003 (2)
C27	0.054 (3)	0.070 (3)	0.069 (3)	0.020 (3)	−0.004 (3)	−0.005 (3)
C21	0.068 (3)	0.063 (3)	0.084 (4)	0.038 (3)	0.003 (3)	−0.008 (3)
O8	0.0413 (15)	0.0492 (16)	0.0402 (17)	0.0146 (13)	0.0076 (13)	0.0058 (13)

### Geometric parameters (Å, °)

**Table:**

Yb1—O7	2.209 (2)	C12—H12B	0.9600
Yb1—O8^i^	2.266 (3)	C12—H12C	0.9600
Yb1—O1	2.340 (3)	C13—C18	1.364 (6)
Yb1—O4	2.360 (3)	C13—C14	1.375 (7)
Yb1—O5	2.369 (2)	C18—H18A	0.9300
Yb1—O2	2.403 (3)	C5—H5A	0.9300
Yb1—N2	2.457 (3)	C10—C11	1.517 (5)
Yb1—N1	2.482 (3)	C32—C31	1.341 (7)
Yb1—C10	2.703 (4)	C32—C38	1.431 (6)
Yb1—C1	2.720 (4)	C32—H32A	0.9300
O1—C1	1.238 (5)	C14—C15	1.371 (8)
O2—C1	1.249 (4)	C14—H14A	0.9300
O3—C4	1.381 (5)	C25—C26	1.357 (8)
O3—C2	1.420 (5)	C25—C24	1.361 (7)
O4—C10	1.257 (4)	C25—H25A	0.9300
O5—C10	1.248 (4)	C24—C23	1.384 (6)
O7—C19	1.252 (4)	C24—H24A	0.9300
O9—C22	1.384 (5)	C26—C27	1.368 (7)
O9—C20	1.415 (4)	C26—H26A	0.9300
O6—C13	1.378 (5)	C2—C3	1.506 (6)
O6—C11	1.418 (5)	C2—H2A	0.9800
N1—C28	1.323 (5)	C15—H15A	0.9300
N1—C36	1.357 (5)	C39—C38	1.406 (5)
N2—C35	1.323 (5)	C39—C36	1.434 (5)
N2—C39	1.365 (4)	C38—C33	1.394 (6)
C1—C2	1.528 (6)	C3—H3A	0.9600
C17—C16	1.357 (8)	C3—H3B	0.9600
C17—C18	1.392 (7)	C3—H3C	0.9600
C17—H17A	0.9300	C34—C33	1.361 (6)
C28—C29	1.405 (6)	C34—C35	1.391 (5)
C28—H28A	0.9300	C34—H34A	0.9300
C8—C7	1.374 (8)	C11—H11A	0.9800
C8—C9	1.394 (7)	C36—C37	1.420 (5)
C8—H8A	0.9300	C30—C29	1.355 (6)
C7—C6	1.355 (7)	C30—C37	1.395 (6)
C7—H7A	0.9300	C30—H30A	0.9300
C19—O8	1.236 (4)	C35—H35A	0.9300
C19—C20	1.528 (5)	C22—C23	1.372 (6)
C16—C15	1.360 (9)	C22—C27	1.384 (6)
C16—H16A	0.9300	C31—C37	1.421 (6)
C4—C9	1.353 (6)	C31—H31A	0.9300
C4—C5	1.374 (6)	C33—H33A	0.9300
C20—C21	1.498 (6)	C29—H29A	0.9300
C20—H20A	0.9800	C23—H23A	0.9300
C6—C5	1.380 (7)	C27—H27A	0.9300
C6—H6A	0.9300	C21—H21A	0.9600
C9—H9A	0.9300	C21—H21B	0.9600
C12—C11	1.509 (6)	C21—H21C	0.9600
C12—H12A	0.9600	O8—Yb1^i^	2.266 (3)
			
O7—Yb1—O8^i^	91.61 (10)	C11—C12—H12C	109.5
O7—Yb1—O1	87.97 (11)	H12A—C12—H12C	109.5
O8^i^—Yb1—O1	149.93 (10)	H12B—C12—H12C	109.5
O7—Yb1—O4	136.78 (9)	C18—C13—C14	121.2 (5)
O8^i^—Yb1—O4	83.17 (10)	C18—C13—O6	124.9 (4)
O1—Yb1—O4	116.77 (11)	C14—C13—O6	114.0 (4)
O7—Yb1—O5	81.63 (9)	C13—C18—C17	118.3 (5)
O8^i^—Yb1—O5	81.00 (9)	C13—C18—H18A	120.8
O1—Yb1—O5	128.51 (9)	C17—C18—H18A	120.8
O4—Yb1—O5	55.16 (9)	C4—C5—C6	120.1 (5)
O7—Yb1—O2	87.71 (10)	C4—C5—H5A	120.0
O8^i^—Yb1—O2	155.75 (10)	C6—C5—H5A	120.0
O1—Yb1—O2	54.30 (9)	O5—C10—O4	121.9 (4)
O4—Yb1—O2	80.83 (10)	O5—C10—C11	117.8 (3)
O5—Yb1—O2	74.91 (9)	O4—C10—C11	120.3 (3)
O7—Yb1—N2	82.59 (10)	O5—C10—Yb1	61.19 (19)
O8^i^—Yb1—N2	74.23 (10)	O4—C10—Yb1	60.76 (19)
O1—Yb1—N2	75.91 (10)	C11—C10—Yb1	178.3 (3)
O4—Yb1—N2	135.43 (9)	C31—C32—C38	121.7 (4)
O5—Yb1—N2	150.11 (10)	C31—C32—H32A	119.1
O2—Yb1—N2	129.56 (10)	C38—C32—H32A	119.1
O7—Yb1—N1	148.62 (10)	C15—C14—C13	119.4 (6)
O8^i^—Yb1—N1	87.15 (10)	C15—C14—H14A	120.3
O1—Yb1—N1	77.99 (11)	C13—C14—H14A	120.3
O4—Yb1—N1	74.20 (9)	C26—C25—C24	120.6 (5)
O5—Yb1—N1	128.89 (9)	C26—C25—H25A	119.7
O2—Yb1—N1	105.73 (11)	C24—C25—H25A	119.7
N2—Yb1—N1	66.89 (10)	C25—C24—C23	120.3 (5)
O7—Yb1—C10	109.09 (11)	C25—C24—H24A	119.9
O8^i^—Yb1—C10	81.95 (11)	C23—C24—H24A	119.9
O1—Yb1—C10	126.41 (11)	C25—C26—C27	120.4 (5)
O4—Yb1—C10	27.69 (10)	C25—C26—H26A	119.8
O5—Yb1—C10	27.50 (10)	C27—C26—H26A	119.8
O2—Yb1—C10	75.42 (11)	O3—C2—C3	106.3 (4)
N2—Yb1—C10	153.85 (11)	O3—C2—C1	110.4 (3)
N1—Yb1—C10	101.78 (11)	C3—C2—C1	110.7 (4)
O7—Yb1—C1	88.88 (11)	O3—C2—H2A	109.8
O8^i^—Yb1—C1	176.88 (10)	C3—C2—H2A	109.8
O1—Yb1—C1	27.01 (10)	C1—C2—H2A	109.8
O4—Yb1—C1	98.56 (11)	C16—C15—C14	120.1 (7)
O5—Yb1—C1	102.11 (11)	C16—C15—H15A	120.0
O2—Yb1—C1	27.35 (10)	C14—C15—H15A	120.0
N2—Yb1—C1	102.79 (11)	N2—C39—C38	121.8 (4)
N1—Yb1—C1	90.83 (11)	N2—C39—C36	118.1 (3)
C10—Yb1—C1	100.80 (12)	C38—C39—C36	120.1 (3)
C1—O1—Yb1	93.9 (2)	C33—C38—C39	118.4 (4)
C1—O2—Yb1	90.6 (2)	C33—C38—C32	123.0 (4)
C4—O3—C2	117.9 (3)	C39—C38—C32	118.6 (4)
C10—O4—Yb1	91.5 (2)	C2—C3—H3A	109.5
C10—O5—Yb1	91.3 (2)	C2—C3—H3B	109.5
C19—O7—Yb1	153.0 (2)	H3A—C3—H3B	109.5
C22—O9—C20	118.8 (3)	C2—C3—H3C	109.5
C13—O6—C11	118.7 (3)	H3A—C3—H3C	109.5
C28—N1—C36	117.9 (3)	H3B—C3—H3C	109.5
C28—N1—Yb1	124.4 (3)	C33—C34—C35	119.0 (4)
C36—N1—Yb1	117.7 (2)	C33—C34—H34A	120.5
C35—N2—C39	117.5 (3)	C35—C34—H34A	120.5
C35—N2—Yb1	124.0 (2)	O6—C11—C12	106.1 (3)
C39—N2—Yb1	118.4 (3)	O6—C11—C10	114.2 (3)
O1—C1—O2	121.0 (4)	C12—C11—C10	109.3 (3)
O1—C1—C2	119.5 (4)	O6—C11—H11A	109.0
O2—C1—C2	119.5 (4)	C12—C11—H11A	109.0
O1—C1—Yb1	59.1 (2)	C10—C11—H11A	109.0
O2—C1—Yb1	62.1 (2)	N1—C36—C37	122.4 (4)
C2—C1—Yb1	176.3 (3)	N1—C36—C39	118.4 (3)
C16—C17—C18	120.4 (6)	C37—C36—C39	119.2 (4)
C16—C17—H17A	119.8	C29—C30—C37	120.0 (4)
C18—C17—H17A	119.8	C29—C30—H30A	120.0
N1—C28—C29	123.1 (4)	C37—C30—H30A	120.0
N1—C28—H28A	118.5	N2—C35—C34	123.9 (4)
C29—C28—H28A	118.5	N2—C35—H35A	118.0
C7—C8—C9	120.6 (6)	C34—C35—H35A	118.0
C7—C8—H8A	119.7	C23—C22—O9	124.6 (4)
C9—C8—H8A	119.7	C23—C22—C27	120.3 (4)
C6—C7—C8	119.5 (5)	O9—C22—C27	115.1 (4)
C6—C7—H7A	120.3	C32—C31—C37	121.2 (4)
C8—C7—H7A	120.3	C32—C31—H31A	119.4
O8—C19—O7	126.0 (3)	C37—C31—H31A	119.4
O8—C19—C20	117.8 (3)	C30—C37—C36	117.2 (4)
O7—C19—C20	116.0 (3)	C30—C37—C31	123.6 (4)
C17—C16—C15	120.6 (6)	C36—C37—C31	119.1 (4)
C17—C16—H16A	119.7	C34—C33—C38	119.3 (4)
C15—C16—H16A	119.7	C34—C33—H33A	120.3
C9—C4—C5	120.3 (5)	C38—C33—H33A	120.3
C9—C4—O3	124.8 (4)	C30—C29—C28	119.2 (4)
C5—C4—O3	114.9 (4)	C30—C29—H29A	120.4
O9—C20—C21	107.7 (3)	C28—C29—H29A	120.4
O9—C20—C19	112.1 (3)	C22—C23—C24	119.0 (5)
C21—C20—C19	109.6 (3)	C22—C23—H23A	120.5
O9—C20—H20A	109.1	C24—C23—H23A	120.5
C21—C20—H20A	109.1	C26—C27—C22	119.4 (5)
C19—C20—H20A	109.1	C26—C27—H27A	120.3
C7—C6—C5	120.3 (5)	C22—C27—H27A	120.3
C7—C6—H6A	119.8	C20—C21—H21A	109.5
C5—C6—H6A	119.8	C20—C21—H21B	109.5
C4—C9—C8	119.2 (5)	H21A—C21—H21B	109.5
C4—C9—H9A	120.4	C20—C21—H21C	109.5
C8—C9—H9A	120.4	H21A—C21—H21C	109.5
C11—C12—H12A	109.5	H21B—C21—H21C	109.5
C11—C12—H12B	109.5	C19—O8—Yb1^i^	138.2 (2)
H12A—C12—H12B	109.5		
			
O7—Yb1—O1—C1	−91.5 (2)	C2—O3—C4—C5	−168.1 (4)
O8^i^—Yb1—O1—C1	178.8 (2)	C22—O9—C20—C21	158.5 (4)
O4—Yb1—O1—C1	51.5 (3)	C22—O9—C20—C19	−80.8 (4)
O5—Yb1—O1—C1	−13.9 (3)	O8—C19—C20—O9	−33.8 (5)
O2—Yb1—O1—C1	−2.9 (2)	O7—C19—C20—O9	151.0 (3)
N2—Yb1—O1—C1	−174.4 (3)	O8—C19—C20—C21	85.8 (4)
N1—Yb1—O1—C1	116.7 (3)	O7—C19—C20—C21	−89.5 (4)
C10—Yb1—O1—C1	20.9 (3)	C8—C7—C6—C5	0.4 (9)
O7—Yb1—O2—C1	92.0 (2)	C5—C4—C9—C8	−1.2 (8)
O8^i^—Yb1—O2—C1	−179.2 (2)	O3—C4—C9—C8	177.8 (5)
O1—Yb1—O2—C1	2.8 (2)	C7—C8—C9—C4	1.2 (8)
O4—Yb1—O2—C1	−129.8 (2)	C11—O6—C13—C18	7.8 (6)
O5—Yb1—O2—C1	173.9 (3)	C11—O6—C13—C14	−172.6 (4)
N2—Yb1—O2—C1	13.6 (3)	C14—C13—C18—C17	−0.4 (7)
N1—Yb1—O2—C1	−59.2 (3)	O6—C13—C18—C17	179.1 (4)
C10—Yb1—O2—C1	−157.6 (3)	C16—C17—C18—C13	0.3 (8)
O7—Yb1—O4—C10	0.6 (3)	C9—C4—C5—C6	0.9 (8)
O8^i^—Yb1—O4—C10	85.7 (2)	O3—C4—C5—C6	−178.3 (5)
O1—Yb1—O4—C10	−118.0 (2)	C7—C6—C5—C4	−0.5 (9)
O5—Yb1—O4—C10	1.9 (2)	Yb1—O5—C10—O4	3.5 (4)
O2—Yb1—O4—C10	−76.0 (2)	Yb1—O5—C10—C11	−178.5 (3)
N2—Yb1—O4—C10	144.9 (2)	Yb1—O4—C10—O5	−3.5 (4)
N1—Yb1—O4—C10	174.6 (2)	Yb1—O4—C10—C11	178.6 (3)
C1—Yb1—O4—C10	−96.9 (2)	O7—Yb1—C10—O5	−2.9 (2)
O7—Yb1—O5—C10	177.2 (2)	O8^i^—Yb1—C10—O5	86.0 (2)
O8^i^—Yb1—O5—C10	−89.8 (2)	O1—Yb1—C10—O5	−105.0 (2)
O1—Yb1—O5—C10	96.6 (2)	O4—Yb1—C10—O5	176.6 (4)
O4—Yb1—O5—C10	−1.9 (2)	O2—Yb1—C10—O5	−85.3 (2)
O2—Yb1—O5—C10	87.3 (2)	N2—Yb1—C10—O5	110.3 (3)
N2—Yb1—O5—C10	−124.0 (3)	N1—Yb1—C10—O5	171.3 (2)
N1—Yb1—O5—C10	−10.9 (3)	C1—Yb1—C10—O5	−95.5 (2)
C1—Yb1—O5—C10	90.2 (2)	O7—Yb1—C10—O4	−179.6 (2)
O8^i^—Yb1—O7—C19	−99.1 (6)	O8^i^—Yb1—C10—O4	−90.6 (2)
O1—Yb1—O7—C19	111.0 (6)	O1—Yb1—C10—O4	78.4 (3)
O4—Yb1—O7—C19	−17.4 (6)	O5—Yb1—C10—O4	−176.6 (4)
O5—Yb1—O7—C19	−18.5 (6)	O2—Yb1—C10—O4	98.1 (2)
O2—Yb1—O7—C19	56.6 (6)	N2—Yb1—C10—O4	−66.3 (4)
N2—Yb1—O7—C19	−173.0 (6)	N1—Yb1—C10—O4	−5.3 (2)
N1—Yb1—O7—C19	173.7 (5)	C1—Yb1—C10—O4	87.9 (2)
C10—Yb1—O7—C19	−17.1 (6)	C18—C13—C14—C15	0.9 (9)
C1—Yb1—O7—C19	84.0 (6)	O6—C13—C14—C15	−178.7 (5)
O7—Yb1—N1—C28	−161.8 (3)	C26—C25—C24—C23	−0.1 (9)
O8^i^—Yb1—N1—C28	109.8 (3)	C24—C25—C26—C27	1.0 (9)
O1—Yb1—N1—C28	−96.5 (3)	C4—O3—C2—C3	−175.8 (4)
O4—Yb1—N1—C28	26.1 (3)	C4—O3—C2—C1	64.2 (5)
O5—Yb1—N1—C28	33.8 (4)	O1—C1—C2—O3	41.3 (5)
O2—Yb1—N1—C28	−49.3 (3)	O2—C1—C2—O3	−139.9 (4)
N2—Yb1—N1—C28	−176.1 (3)	O1—C1—C2—C3	−76.1 (5)
C10—Yb1—N1—C28	28.7 (3)	O2—C1—C2—C3	102.6 (5)
C1—Yb1—N1—C28	−72.5 (3)	C17—C16—C15—C14	1.1 (12)
O7—Yb1—N1—C36	20.3 (4)	C13—C14—C15—C16	−1.2 (11)
O8^i^—Yb1—N1—C36	−68.1 (3)	C35—N2—C39—C38	0.9 (6)
O1—Yb1—N1—C36	85.6 (3)	Yb1—N2—C39—C38	−175.8 (3)
O4—Yb1—N1—C36	−151.8 (3)	C35—N2—C39—C36	−177.7 (3)
O5—Yb1—N1—C36	−144.1 (2)	Yb1—N2—C39—C36	5.5 (4)
O2—Yb1—N1—C36	132.8 (3)	N2—C39—C38—C33	−1.6 (6)
N2—Yb1—N1—C36	6.0 (2)	C36—C39—C38—C33	177.0 (4)
C10—Yb1—N1—C36	−149.2 (3)	N2—C39—C38—C32	178.4 (4)
C1—Yb1—N1—C36	109.6 (3)	C36—C39—C38—C32	−3.0 (6)
O7—Yb1—N2—C35	5.1 (3)	C31—C32—C38—C33	−179.0 (5)
O8^i^—Yb1—N2—C35	−88.7 (3)	C31—C32—C38—C39	1.0 (7)
O1—Yb1—N2—C35	94.8 (3)	C13—O6—C11—C12	170.1 (3)
O4—Yb1—N2—C35	−151.2 (3)	C13—O6—C11—C10	−69.4 (4)
O5—Yb1—N2—C35	−53.6 (4)	O5—C10—C11—O6	160.0 (3)
O2—Yb1—N2—C35	85.9 (3)	O4—C10—C11—O6	−21.9 (5)
N1—Yb1—N2—C35	177.6 (3)	O5—C10—C11—C12	−81.3 (4)
C10—Yb1—N2—C35	−113.9 (4)	O4—C10—C11—C12	96.7 (4)
C1—Yb1—N2—C35	92.2 (3)	C28—N1—C36—C37	−3.9 (6)
O7—Yb1—N2—C39	−178.4 (3)	Yb1—N1—C36—C37	174.2 (3)
O8^i^—Yb1—N2—C39	87.8 (3)	C28—N1—C36—C39	176.2 (4)
O1—Yb1—N2—C39	−88.7 (3)	Yb1—N1—C36—C39	−5.7 (4)
O4—Yb1—N2—C39	25.3 (3)	N2—C39—C36—N1	0.2 (5)
O5—Yb1—N2—C39	122.9 (3)	C38—C39—C36—N1	−178.4 (3)
O2—Yb1—N2—C39	−97.6 (3)	N2—C39—C36—C37	−179.7 (3)
N1—Yb1—N2—C39	−5.9 (2)	C38—C39—C36—C37	1.6 (6)
C10—Yb1—N2—C39	62.6 (4)	C39—N2—C35—C34	0.4 (6)
C1—Yb1—N2—C39	−91.3 (3)	Yb1—N2—C35—C34	177.0 (3)
Yb1—O1—C1—O2	5.2 (4)	C33—C34—C35—N2	−1.0 (7)
Yb1—O1—C1—C2	−176.1 (3)	C20—O9—C22—C23	−0.5 (6)
Yb1—O2—C1—O1	−5.1 (4)	C20—O9—C22—C27	−179.1 (4)
Yb1—O2—C1—C2	176.2 (3)	C38—C32—C31—C37	2.4 (8)
O7—Yb1—C1—O1	87.7 (3)	C29—C30—C37—C36	1.8 (6)
O4—Yb1—C1—O1	−135.0 (2)	C29—C30—C37—C31	−177.9 (4)
O5—Yb1—C1—O1	168.9 (2)	N1—C36—C37—C30	2.0 (6)
O2—Yb1—C1—O1	174.9 (4)	C39—C36—C37—C30	−178.1 (4)
N2—Yb1—C1—O1	5.6 (3)	N1—C36—C37—C31	−178.2 (4)
N1—Yb1—C1—O1	−60.9 (3)	C39—C36—C37—C31	1.7 (6)
C10—Yb1—C1—O1	−163.0 (2)	C32—C31—C37—C30	176.0 (5)
O7—Yb1—C1—O2	−87.2 (2)	C32—C31—C37—C36	−3.8 (7)
O1—Yb1—C1—O2	−174.9 (4)	C35—C34—C33—C38	0.3 (7)
O4—Yb1—C1—O2	50.1 (2)	C39—C38—C33—C34	0.9 (6)
O5—Yb1—C1—O2	−6.0 (3)	C32—C38—C33—C34	−179.1 (4)
N2—Yb1—C1—O2	−169.3 (2)	C37—C30—C29—C28	−3.6 (7)
N1—Yb1—C1—O2	124.2 (2)	N1—C28—C29—C30	1.7 (7)
C10—Yb1—C1—O2	22.0 (3)	O9—C22—C23—C24	−174.9 (4)
C36—N1—C28—C29	2.0 (6)	C27—C22—C23—C24	3.6 (7)
Yb1—N1—C28—C29	−175.9 (3)	C25—C24—C23—C22	−2.2 (8)
C9—C8—C7—C6	−0.8 (9)	C25—C26—C27—C22	0.3 (8)
Yb1—O7—C19—O8	102.6 (6)	C23—C22—C27—C26	−2.7 (7)
Yb1—O7—C19—C20	−82.6 (6)	O9—C22—C27—C26	176.0 (4)
C18—C17—C16—C15	−0.7 (10)	O7—C19—O8—Yb1^i^	−7.7 (6)
C2—O3—C4—C9	12.8 (6)	C20—C19—O8—Yb1^i^	177.6 (2)

Symmetry codes: (i) −*x*+1, −*y*+1, −*z*.
